# Inhibition of the STAT3 target SGK1 sensitizes diffuse large B cell lymphoma cells to AKT inhibitors

**DOI:** 10.1038/s41408-019-0203-y

**Published:** 2019-03-29

**Authors:** Li Lu, Fen Zhu, Yangguang Li, Shuichi Kimpara, Nguyet Minh Hoang, Sheida Pourdashti, Lixin Rui

**Affiliations:** 1 Department of Medicine, University of Wisconsin School of Medicine and Public Health, Madison, WI 53726 USA; 2 Carbone Cancer Center, University of Wisconsin School of Medicine and Public Health, Madison, WI 53726 USA

Diffuse large B cell lymphoma (DLBCL) is the most common non-Hodgkin lymphoma, including two main molecular subtypes termed as activated B-cell-like (ABC) and germinal center B-cell-like (GCB)^1^. ABC DLBCL shares gene expression signatures with activated B cells and STAT3 is a critical transcriptional regulator of this subtype^1^. To investigate the gene regulation mechanism by STAT3 in ABC DLBCL cells and activated B cells, we performed ChIP-seq analysis. We treated ABC DLBCL cell line TMD8 with the JAK1/2 inhibitor AZD1480 that inhibits STAT3 phosphorylation as a control^1^. We used phospho-STAT3 antibody to increase specificity of STAT3 binding. Using the model-based analysis of ChIP-seq (MACS) for peak calling, we identified a total of 7470 STAT3 binding sites (peaks) in TMD8 cells when compared with the AZD1480-treated control sample (Fig. 1a, Supplemental Table 1). More than 60% of peaks are present in the promoter, upstream enhancer, and gene body regions (Fig. 1b). Specificity of these STAT3 binding sites was confirmed by the MEME motif enrichment analysis (Fig. 1c).

**Fig. 1:**
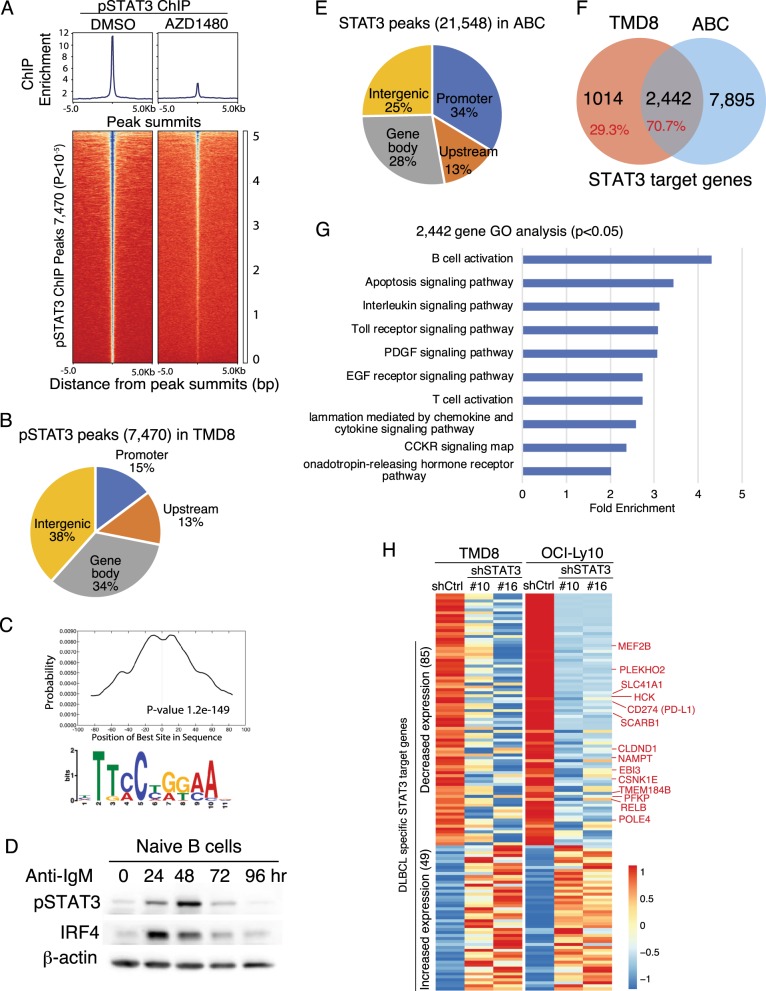
Genome-wide analysis of STAT3 target genes in TMD8 cells and activated B cells. **a** Heat maps of pSTAT3 ChIP-seq in TMD8 cells, after 4 h treatment with either DMSO or 4 μM AZD1480. pSTAT3 peak summits were centered with 5 kb of flanking sequence either side. Blue color indicates higher density of reads. pSTAT3 peaks were ranked by signal intensity at the peak center, and the same order was used to display the AZD1480 treated sample. **b** pSTAT3 peaks show a major distribution in the gene promoter (±1 Kb to TSS), upstream enhancer (−15 Kb to TSS) and gene body. **c** The CentriMo plot shows the distribution of known STAT3 motif in the ChIP-seq peak summit regions (*p* < 0.001). **d** Immunoblot analysis of pSTAT3 and IRF4 in anti-IgM (10 μg/ml) stimulated naive B cells. β-actin served as a loading control. **e** STAT3 ChIP-seq peaks in normal activated B cells show a major distribution in the gene promoter (± 1Kb to TSS), upstream enhancer (−15 Kb to TSS) and gene body. **f** Venn diagram shows 2441 genes shared in pSTAT3 ChIP-seq in TMD8 cells and STAT3 ChIP-seq in activated B cells (ABC) and 1014 genes specific for TMD8 cells. **g** Gene ontology analysis of 2442 STAT3 common target genes between TMD8 and activated B cells (*p* < 0.05). **h** Heat maps show mRNA levels of pSTAT3 binding genes after knockdown of STAT3 in TMD8 cells (Data from GSE106844)

Stimulation of the B cell receptor (BCR) can activate STAT3 in lymphoma cells^2^. To test whether this is the case in naive B cells, we stimulated peripheral blood B cells with anti-IgM antibody. Indeed, we detected STAT3 phosphorylation after 24 h treatment with a peak at 48 h (Fig. 1d). B cell activation was confirmed by IRF4, a downstream effector of BCR signaling (Fig. 1d). Then, we used 24 h-stimulated peripheral blood B cells for STAT3 ChIP-seq analysis and identified a total of 21,548 STAT3 binding sites (peaks) when compared with the input control (Fig. 1e, Supplemental Table 1). We observed 75% of peaks present in the promoter, upstream enhancer, and gene body regions (Fig. 1b).

Based on genomic loci of these peaks, we mapped individual genes within a window extending from −15 kilobases (kb) 5′ of the transcriptional start site (TSS) to the 3′ end of any annotated transcript associated with the gene, as for our previous study^1^. We identified 3456 potential STAT3 target genes in TMD8 cells and 10,337 in activated B cells, with an overlap of 2442 genes between TMD8 and activated B cells (Fig. 1f, Supplemental Table 1). Considering these overlapped genes as common STAT3 targets in normal and malignant cells, we performed PANTHER gene ontology analysis. The results revealed that these common target genes were enriched for biological processes that include B cell activation, apoptosis, cytokine signaling, EGF/PDGF signaling, Toll receptor signaling, and inflammation (Fig. 1g). Consistent with our previous study^1^, these common STAT3 target genes include STAT3 itself, the type I interferon pathway genes (STAT1, STAT2, IRF7, IRF9), NFκB genes (NFκB2, NFκBIA, NFκBIZ), and apoptosis pathway genes (BCL2, MCL1, BCL2L11, CASP8) (Fig. S1). Most of these STAT3 target genes change their expression in ABC DLBCL cells, based on our previous RNA-seq analysis (Fig. S2, Supplemental Table 1)^1^. Taken together, the data suggest an important role for STAT3 in the pathogenesis of ABC DLBCL, as well as in the normal immune response.

The above STAT3 ChIP-seq analysis also revealed 1014 genes that are ABC DLBCL specific (Fig. 1f). Among them, 85 genes reduced their expression while the expression of 49 genes was increased after STAT3 knockdown (Fig. 1h, Supplemental Table 1). Some of these STAT3 target genes are highly expressed and significantly contribute to DLBCL biology. MEF2B, a transcriptional activator, directly activates BCL6 in normal germinal center B cells and is required for DLBCL proliferation^3^. Expression and activation of hematopoietic cell kinase (HCK) is induced due to activating mutations in MYD88 that are present in ∼40% of ABC DLBCL^4^. HCK activity promotes the survival and proliferation of ABC DLBCL cells by enhancing BTK, PI3K/AKT, and MAP kinase signaling in mutated MYD88 ABC DLBCL cells^4^.

In addition, the tumor specific STAT3 target genes include those that are involved in immune regulation and cell metabolism, such as CD274 (PD-L1) and the high-affinity HDL receptor, scavenger receptor type B1 (SCARB1) (Fig. 1h, Fig. S3). PD-L1, an immune checkpoint molecule, is overexpressed in ~25% of non-GCB DLBCL but rarely expressed in GCB DLBCL^5^. PD-L1 expression on DLBCL cells is associated with poorer overall survival^5^. Recent studies suggest that PD-L1 overexpression can result from genomic amplifications and translocations, and BCR-mediated NFATc1 activation through IL-10/STAT3^5,6^. In support of these findings, our genome-wide analysis revealed that PD-L1 is a direct target gene of STAT3 in ABC DLBCL cells (Fig. 1h, Fig. S3). PD-L1 expression allows ABC DLBCL cells to escape the immune surveillance of tumor-specific cytotoxic T cells^5^. Therefore, the above and other STAT3 tumor specific target genes identified from the study can be used for the development of a targeted therapeutic strategy in DLBCL.

Serum-regulated and glucocorticoid-regulated kinase 1 (SGK1), a serine/threonine kinase of the AGC kinase family^7,8^, is a STAT3 binding gene in ABC DLBCL and activated B cells (Fig. 2a). Our previous RNA-seq analysis revealed that SGK1 expression decreased after STAT3 knockdown in ABC DLBCLs (Fig. 2b). Notably, SGK1 expression was induced by anti-IgM antibody in activated B cells (Fig. 2c) in which STAT3 is activated (Fig. 1d). As expected, the level of SGK1 protein expression was reduced after STAT3 inhibition by the JAK1/2 inhibitor AZD1480 (Fig. 2d) while increased by overexpression of the constitutively activated form of STAT3 (STAT3-C), with activating mutations (A661C and N663C) in the SH2 domain (Fig. 2e)^1^. These data suggest that STAT3 directly regulates SGK1 expression in both ABC DLBCL and activated B cells.

**Fig. 2:**
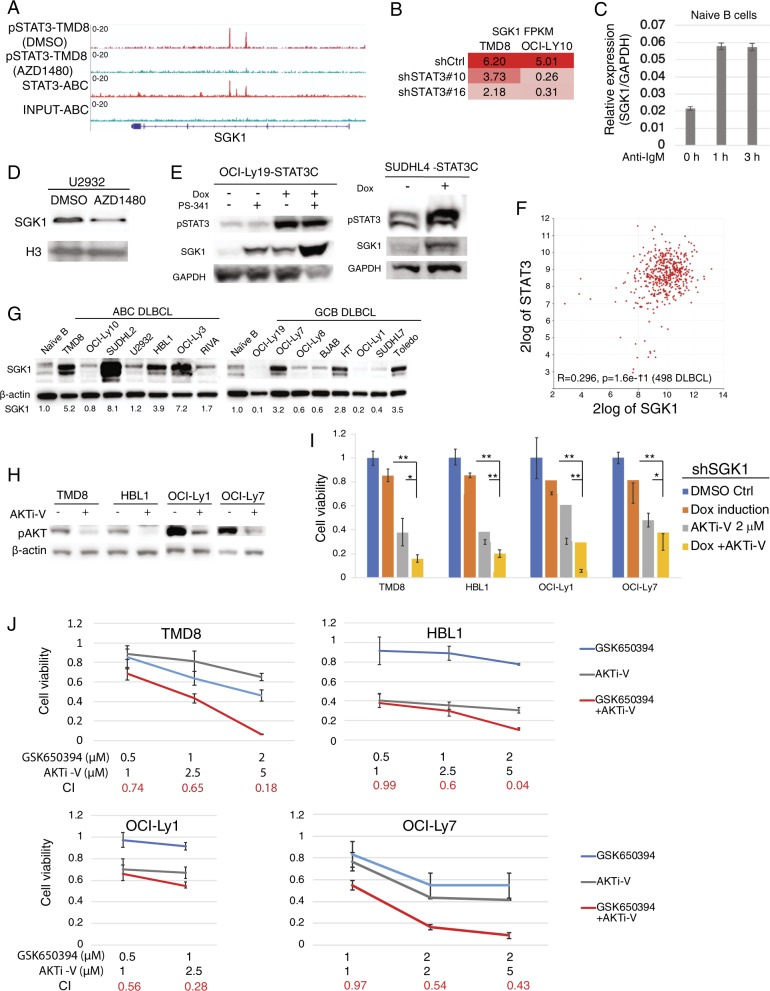
Inhibition of the STAT3 target SGK1 sensitizes DLBCL cells to AKT inhibitors. **a** STAT3 is recruited to regulatory regions of SGK1. TMD8 DMSO controls or activated B cell samples in red, AZD1480-treated TMD8 samples or activated B cell input samples in green. **b** RNA-seq FPKM shows a reduction in SGK1 expression after knockdown of STAT3 in TMD8 and OCI-Ly10 cells (Data from GSE106844). **c** Real-time PCR shows elevated SGK1 expression in naive B cells upon anti-IgM (10 μg/ml) stimulation. Error bars represent mean ± of SE of triplicates. **d** Immunoblot shows reduced SGK1 protein levels in U2932 cells treated with AZD1480 (4 μM, 12 h). **e** Immunoblot analysis of indicated proteins in OCI-Ly19 and SUDHL4 after 2 days of retroviral expression of the constitutively activated STAT3 (STAT3-C), combined with or without 4 h treatment of the proteasome inhibitor PS-341 (250 nM). **f** SGK1 expression is positively correlated with STAT3 expression in 498 patient samples. Data were generated from R2: Genomics Analysis and Visualization Platform (https://hgserver1.amc.nl/cgi-bin/r2/main.cgi). **g** Immunoblot analysis of SGK1 protein levels in naive B cells, ABC, and GCB DLBCL cell lines. β-actin served as a loading control. **h** Immunoblot analysis of pAKT protein levels in TMD8, HBL, OCI-Ly1, and OCI-Ly7 cells after 24 h treatment of the AKT inhibitor AKTi-V (20 μM). **i** CellTiter-Glo™ Luminescent Cell Viability Assay of TMD8, HBL1, OCI-Ly1, and OCI-Ly7 cells after 6 days induction with SGK1 knockdown (shSGK1#2) or 6 days treatment of AKTi-V (2 μM), or both. Data indicate mean ± SE of triplicates. *p < 0.05; **p < 0.01. **j** CellTiter-Glo™ Luminescent Cell Viability Assay of TMD8, HBL1, OCI-Ly1, and OCI-Ly7 cells after 6 days treatment of the indicated concentrations of AKTi-V or the SGK1 inhibitor GSK650394, or both. Data indicate mean ± SE of triplicates. Combination index (CI) was calculated with CompuSyn software. CI < 1 indicates synergy

JAK1/STAT3 signaling is activated in ABC DLBCL but not in the GCB subtype^1^. The mechanism of SGK1 expression in GCB DLBCL remains unknown. Recent next generation sequencing studies demonstrate that SGK1 is mutated in 10–13% of GCB DLBCL but rarely in ABC DLBCL^9^. Most concurrent mutations are located on the N-terminus (1–98) of SGK1 (Fig. S4A, B)^9^, which contributes to its constitutive degradation by the ubiquitin-proteasome pathway (Fig. S4C)^10^. To test whether the N-terminal mutations prevent proteasomal degradation, we selected three most concurrent mutations (A26V, A48V, H51P) from the 1001 patient database^9^ for protein turnover analysis. We expressed these mutants in parallel with SGK1 wild-type (isoform 1; NM_005627.3) in 293T cells. SGK1 isoform 3 (NM_001143677.1) served as a control as it has a different N-terminal sequence that does not mediate proteasomal degradation. We found that, unlike stabilized SGK1 isoform 3, all three SGK1 mutants and wild-type protein (SGK1-ISO1) were expressed at a lower level in the DMSO control but increased after proteasomal inhibition by PS-341 (Fig. S4D), suggesting the SGK1 mutations do not stabilize the protein. A similar result was observed for SGK1 endogenous protein both in several SGK1 wild-type cell lines and in OCI-Ly1 cell line that harbors SGK1 N70K mutation (Fig. S4B–E). Given that SGK1 mutations are frequent and specific in the GCB subtype, the function of mutant SGK1 would be worth investigating further.

In recent years, PI3K inhibitors have emerged as a targeted therapy in DLBCL^11^. However, their use has been limited by some of their associated adverse events^11^. The development of combination therapies is necessary to overcome drug resistance and to minimize overlapping toxicities. In DLBCL, SGK1 was highly expressed and its expression was correlated with STAT3 expression (Fig. 2f). Varied levels of SGK1 expression were observed in DLBCL cell lines as well (Fig. 2g). To test whether SGK1 expression is essential for cell survival, we generated two different shRNAs against SGK1 (Fig. S5A) and expressed them in 5 ABC and 8 GCB DLBCL cell lines (Fig. S5B). After 12 days of shSGK1 expression, none of these cell lines were sensitive except two ABC DLBCL cell lines TMD8 and SUDHL2 that showed a moderate sensitivity (Fig. S5B). The similar result was obtained using the SGK1 inhibitor GSK650394 (Fig. S5C)^12^.

Since both AKT and SGK1 are expressed and activated in ABC and GCB DLBCL cells^13^, which have the ability to engage in opportunistic compensation when one of the two kinases is genetically repressed or pharmacologically inhibited^8^, we hypothesized that inhibiting SGK1 enhances anti-tumor effects of AKT inhibitors in DLBCL. To test this hypothesis, we used AKT inhibitor V^14^ to treat two ABC DLBCL cell lines (TMD8 and HBL1) and two GCB DLBCL cell lines (OCI-Ly1 and OCI-Ly7), all of which expressed shSGK1#2. Immunoblot analysis confirmed a reduction in AKT phosphorylation by the drug (Fig. 2h). Indeed, knockdown of SGK1 increased the cytotoxicity of AKT inhibitor V in all four cell lines (Fig. 2i). We found a similar result using another AKT inhibitor AZD5363 (Fig. S6A)^15^. Notably, synergistic cell killing was observed using the two kinase inhibitors (Fig. 2j, Fig. S6B). Cell viability was not significantly reduced by the treatment of the SGK1 inhibitor alone except for TMD8 cells, which showed a moderate sensitivity. Therefore, our study suggests that co-targeting SGK1 and AKT is more effective, which can be considered a potential therapeutic strategy for the treatment of DLBCL.

## Supplementary information

Supplemental Materials

Supplemental Table 1
