# Supplementary material for: Inhibition of the STAT3 target SGK1 sensitizes diffuse large B cell lymphoma cells to AKT inhibitors
Source: Blood Cancer J. 2019 Mar 29;9(4):43. doi: 10.1038/s41408-019-0203-y (PMC6441016; doi:10.1038/s41408-019-0203-y)
Supplement: Supplementary file 1 — Supplemental Materials [file 41408_2019_203_MOESM1_ESM.pdf]

Supplemental Materials

Inhibition of the STAT3 target SGK1 sensitizes diffuse large B cell lymphoma cells to AKT inhibitors

Li Lu[1,2], Fen Zhu[1,2], Yangguang Li[1,2], Shuichi Kimpara[1,2], Nguyet Minh Hoang[1,2], Sheida Pourdashti[1] and Lixin Rui[1,2*]

[1]Department of Medicine and [2]Carbone Cancer Center, University of Wisconsin School of Medicine and Public Health, Madison, WI, USA.

[*]*Corresponding Author*
Lixin Rui
1111 Highland Ave.
Madison, WI 53705
USA
lrui@medicine.wisc.edu
Phone: (608) 265-8525
Fax: (608) 262-4598

Materials and Methods

Cell lines and culture

All doxycycline-inducible human diffuse large B cell lymphoma cell lines that express the bacterial tetracycline repressor were engineered as described previously [1]. Doxycycline (20 ng/mL) was used for inducing the expression of genes of interest. The cell lines were grown in RPMI 1640 media (HyClone) supplemented with 10% FBS (Atlanta Biologicals), 100 U/mL penicillin, 100 µg/mL streptomycin (Corning Cellgro), 2 mM GlutaGRO (Corning Cellgro), 1×MEM-NEAA (Quality Biological, Inc.), and 1 mM Sodium Pyruvate Solution (HyClone). All cultures were routinely tested for mycoplasma contamination. Human embryonic kidney cell line 293T was cultured in DMEM (HyClone) with 10% FBS. All cell lines were cultured at 37 °C in a 5% CO_2 atmosphere.

Naive B cell isolation

Naïve B cell was isolated from PBMC (peripheral blood mononuclear cells, collected with the protocol #2013-1570 approved by the University of Wisconsin-Madison institutional review boards) using naïve B cell isolation kit (Miltenyi Biotec) according to the manufacturer's instructions. Briefly, PBMC was isolated by density gradient centrifugation, and blood cells were lysed with ACK lysing buffer. Naïve B cell biotin-antibody cocktail was then added and incubated for 5 min at 4 °C. After that, anti-biotin microbeads were added and incubated for additional 10 min at 4 °C. Negative selection was then performed by MACS Pro machine. The purity was measured by FITC-CD20 (Miltenyi Biotech LT20) and PE-CD27 (Ebiosciences O323) staining (~98%). Anti-IgM was purchased from Southern biotech (#2020-01).

Plasmid construction and retroviral transduction

The SGK1 full length/ truncated fragments were amplified from cDNA of TMD8. The following primers were used: SGK1-ISO1-FLAG forward (5'-

CCCAAGCTTATGGACTACAAGGACGACGATGACAAGATGACGGTGAAAACTGAGG-3’);
SGK1-ISO3-FLAG forward (5’-
CCCAAGCTTATGGACTACAAGGACGACGATGACAAGATGTCATCTCAGAGTTCCAG-3’);
SGK1-Δ(1-98) -FLAG forward (5’-
CCCAAGCTTATGGACTACAAGGACGACGATGACAAGATGTTTCACTTCTTGAAAG-3’);
SGK1-END reverse (5’- CCGCTCGAGCAAGCCCTAACAGGGTTCAG-3’).
SGK1 single mutations were engineered by GeneArt® Site-Directed Mutagenesis System (Invitrogen #A13282); The STAT3-C (www.addgene.org/24983/) was verified with the A661C and N663C mutations. SGK1 and STAT3-C fragments were subcloned into the pRetroCMV/TO-PG vector, which is an inducible CMV/TO retroviral vector with the selection marker puromycin [1]. shRNAs were prepared in a doxycycline-inducible retroviral vector pRSMX-PG with the marker EGFP and the selection marker puromycin as described previously [1], with sequences of shSGK1#1 (5’-GGCAGAAGAAGTGTTCTAT-3’) and shSGK1#2 (5′-GCCGAAACACAGCTGAAAT-3’).
For pRetroCMV/TO-PG or pRSMX-PG constructs, the mutant ecotropic envelope-expressing plasmid pHIT/EA6 × 3*, and the gag-pol expressing plasmid pHIT60 were used to transfect the 293T cells using the Genejuice transfection reagent (Millipore; #70967-3) as described previously [1]. The doxycycline-inducible lymphoma cell lines were infected by retroviral supernatants in the presence of 8 μg/mL Polybrene.

Cell viability assay
Cells collected over 3-12 days after the addition of 20 ng/ml doxycycline were analyzed with a MACSQuant Analyzer 10 Flow Cytometer (Miltenyi Biotec). GFP+ cell populations were analyzed using FlowJo software (BD Biosciences) for shRNA-expressing cells as compared to a control shRNA (shSC4) [2].

For drug related cell viability experiments, cells were seeded in 96-well plates with each well containing 2,000 cells in 100μl of media with drug, fresh media and drug were added again at day 3. Each treatment was carried out in triplicates with control treatment by DMSO. Cell viability was scored by CellTiter GLO 2.0 (Promega #G9242) following the instructions. Luminescence was measured by ENSPIRE (PerkinElmer) plate reader. AZD1480, GSK650394 and Triciribine (ATKi-V) were purchased from Selleck Chemicals (S2162; S7209; S1117). AZD5363 was purchased from Cayman chemical (1143532-39-1). Statistical analyses were performed by using Prism 6 for Mac OS X. Student *t* test were used. Significant differences were indicated by * $p \leq 0.05$, ** $p \leq 0.01$.

Apoptosis analysis. Cell apoptosis was measured using FITC (Fluorescein isothiocyanate)-Annexin V and PI (propidium iodide) staining kit (BD Pharmingen# 556547) following the manufacture’s protocols. Briefly, cells with different treatments were collected and washed twice with cold PBS, and then resuspended in 1×binding buffer from the kit. FITC-Annexin V and PI were added and incubated for 15 min at room temperature in the dark before flow cytometry. Data were analyzed by Flowjo software.

Immunoblot assay
Cells were lysed using MAPK lysis buffer (50 mM HEPES, 4 mM sodium pyrophosphate, 10 mM sodium fluoride, 2 mM orthovanadate, 100 mM NaCl, 10 mM EDTA, pH 7.5) with protease inhibitor cocktail (Sigma #P8340). Protein concentrations were determined by BCA assay (Thermo Scientific). Proteins were separated by 4-12% SDS-PAGE gel (Expedeon Inc #nxg41212) and transferred onto nitrocellulose membranes (BioRad #1620115). After blocking with nonfat milk, the membrane was probed with primary antibodies against proteins of interest.

The antibodies used in this study: Phospho-STAT3(Tyr-705) (Cell Signaling #9145); β-actin (Cell Signaling #4967); SGK1 (Cell Signaling #12103); FLAG (Sigma F1804); IRF4 (Santa Cruz sc-28696); GAPDH (Cell Signaling #3683); H3 (Cell Signaling #4620); pAKT (Cell Signaling #9271).

Quantitative real-time PCR (qPCR)
Total RNA was extracted using RNeasy Plus Mini Kit (Qiagen) according to the manufacture's protocol. 3 μg RNA was reverse transcribed with SuperScript IV Reverse Transcriptase (ThermoFisher # 18090050). qPCR was performed using an ABI Stepone Plus Real-Time PCR System. Primers used in this study were the following: SGK1-forward (5'-AGGGCAGTTTTGGAAAGGTT-3'); SGK1-reverse (5'-CTGTAAAACTTTGACTGCATAGAACA-3'); GAPDH-Forward (5'-GGGAAACTGTGGCGTGAT-3'); GAPDH-Reverse (5'-GAGTGGGTGTCGCTGTTGA-3').

Chromatin immunoprecipitation (ChIP) and sequencing
Chromatin immunoprecipitation experiments were performed as previously described [2]. Briefly, cells were treated by DMSO or AZD1480 (4μM for 4 h) in parallel, after treatment, ten million cells were crosslinked with formaldehyde (1%) for 10 minutes at room temperature, followed by the addition of glycine (125mM) for 5 minutes. The nuclear pellets were collected and sonicated with S220 focused-ultrasonicators (Covaris). Then STAT3 antibody (Santa Cruz sc-482X) or pSTAT3 antibody (Cell Signaling #9145) was added and incubated at 4°C for 16 h on a rotator. Dynabeads protein A was pre-washed and added for 2 h incubation in 4°C on a rotator. The immunoprecipitated complex was eluted in elution buffer (1% SDS, 0.1M NaHCO3) by 65°C vortex for 5 mins. DNA was purified using Qiagen PCR purification kit according to manufacturer's protocol. 10ng of ChIPed DNA was used to generate ChIP-seq library using Ovation Ultralow Library System V2 (NuGen Technologies), according to the manufacturer's protocol. Sequencing was performed on Illumina Hiseq 2500 (50bp length) at University of Wisconsin Biotechnology Center DNA sequencing facility. Data are available at NCBI GEO (GSE123398).

Data analysis
For ChIPseq analysis, raw reads were mapped to the human reference genome (hg19) by Bowtie2 (V2.1.0) using default parameters. Identical reads were collapsed into one. ChIP-seq data were visualized in Integrative Genomic Viewer (V2.3 40). Peak was called by Model-based Analysis of ChIP-seq (MACS) (V1.4.2) with $p<10^{-5}$. Annotated genes with its 15Kb upstream that overlapped with the peaks were considered binding gene. ±100 bp around a peak summit was used for motif discovery. MEME suite was used for motif discovery (http://meme-suite.org/). ChIP heatmaps were generated by deepTools [3]. The model-based analysis of ChIP-seq (MACS) was performed for peak calling [4]. Gene ontology analysis was launched by PANTHER classification system (http://pantherdb.org/).

References

1. Ngo VN, Davis RE, Lamy L, Yu X, Zhao H, Lenz G*, et al.* A loss-of-function RNA interference screen for molecular targets in cancer. *Nature* 2006 May 4; **441**(7089)**:** 106-110.

2. Rui L, Drennan AC, Ceribelli M, Zhu F, Wright GW, Huang DW*, et al.* Epigenetic gene regulation by Janus kinase 1 in diffuse large B-cell lymphoma. *Proceedings of the National Academy of Sciences of the United States of America* 2016 Nov 15; **113**(46)**:** E7260-E7267.

3. Ramirez F, Ryan DP, Gruning B, Bhardwaj V, Kilpert F, Richter AS*, et al.* deepTools2: a next generation web server for deep-sequencing data analysis. *Nucleic Acids Res* 2016 Jul 8; **44**(W1)**:** W160-165.

4. Zhang Y, Liu T, Meyer CA, Eeckhoute J, Johnson DS, Bernstein BE*, et al.* Model-based analysis of ChIP-Seq (MACS). *Genome Biol* 2008; **9**(9)**:** R137.

Supplemental Figures

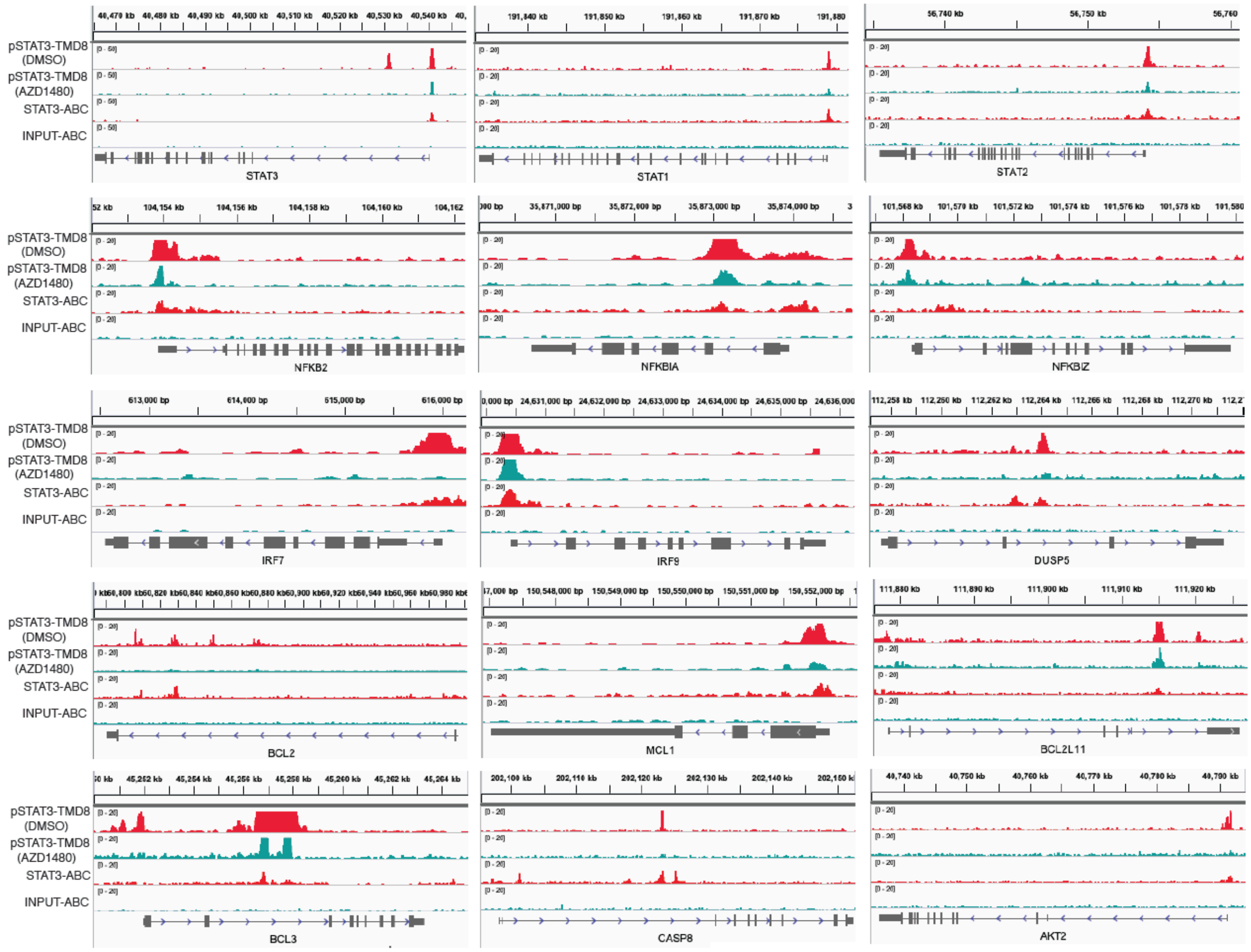

Figure S1. Representative STAT3 common target genes between TMD8 and activated B cells. Shown are read density tracks of representative common STAT3 target genes (TMD8 DMSO controls or activated B cell samples in red, AZD1480 treated TMD8 samples or activated B cell input samples in green).

A

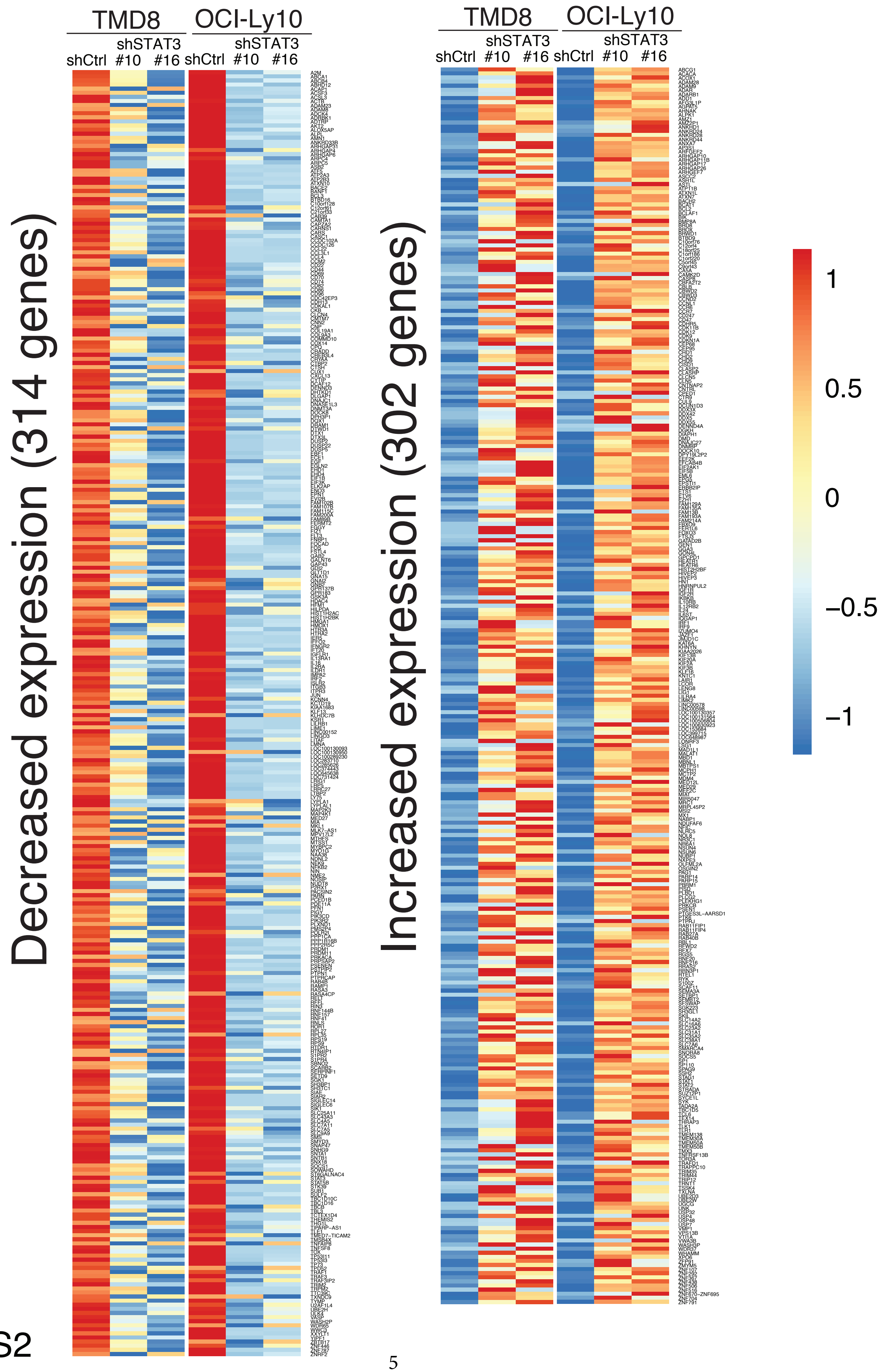
TMD8
OCI-Ly10
shSTAT3
shCtrl
#10
#16
TMD8
OCI-Ly10
shSTAT3
shCtrl
#10
#16
Decreased expression (314 genes)
Increased expression (302 genes)
1
0.5
0
−0.5
−1

Figure S2

Figure S2. Expression of STAT3 common target genes between TMD8 and activated B cells. (A) Heat maps show mRNA levels of pSTAT3 binding genes after knockdown of STAT3 in TMD8 cells (Data from GSE106844).

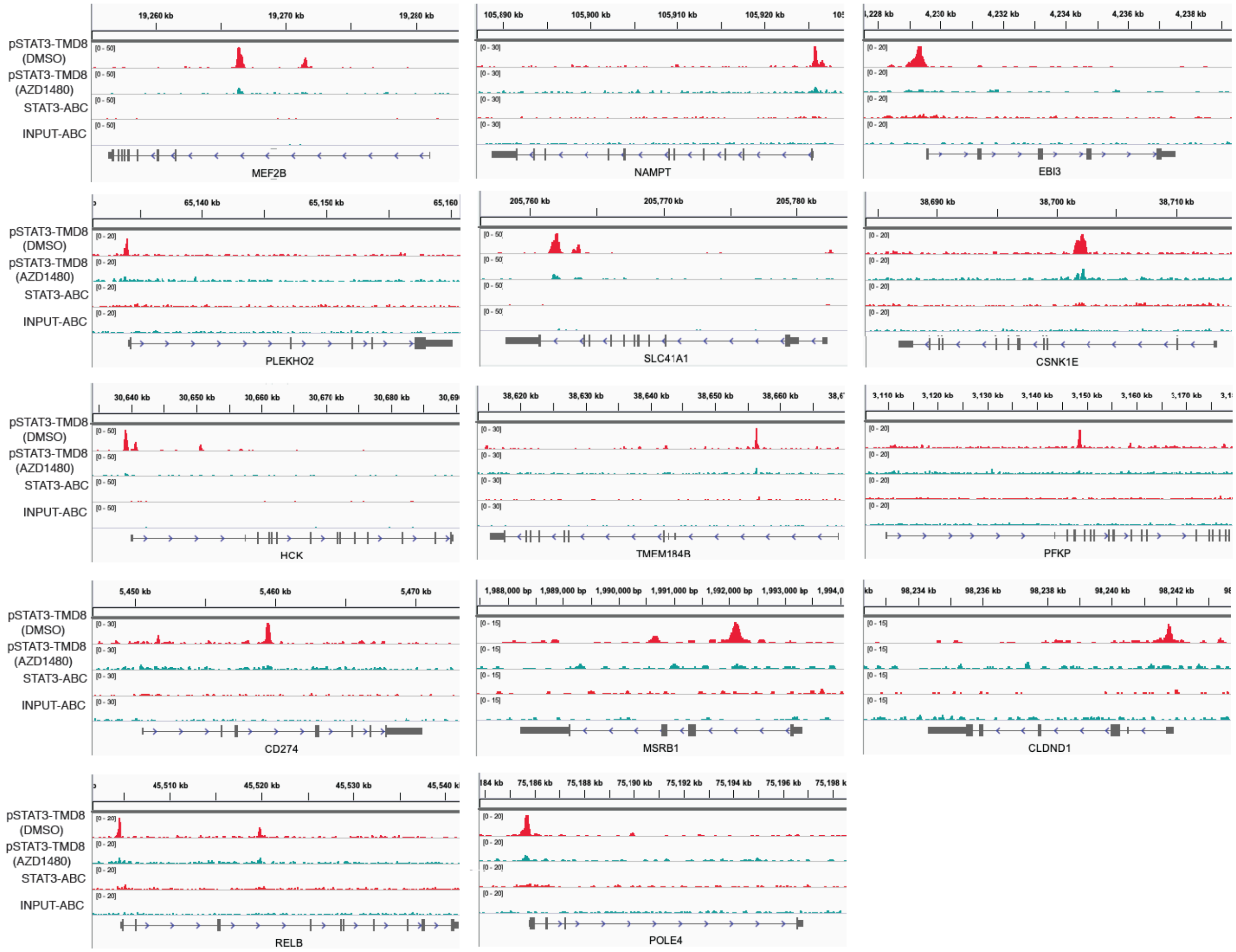

Figure S3. Representative STAT3 target genes specific for TMD8 cells. Shown are read density tracks of representative tumor specific STAT3 target genes (TMD8 DMSO controls or activated B cell samples in red, AZD1480 treated TMD8 samples or activated B cell input samples in green).

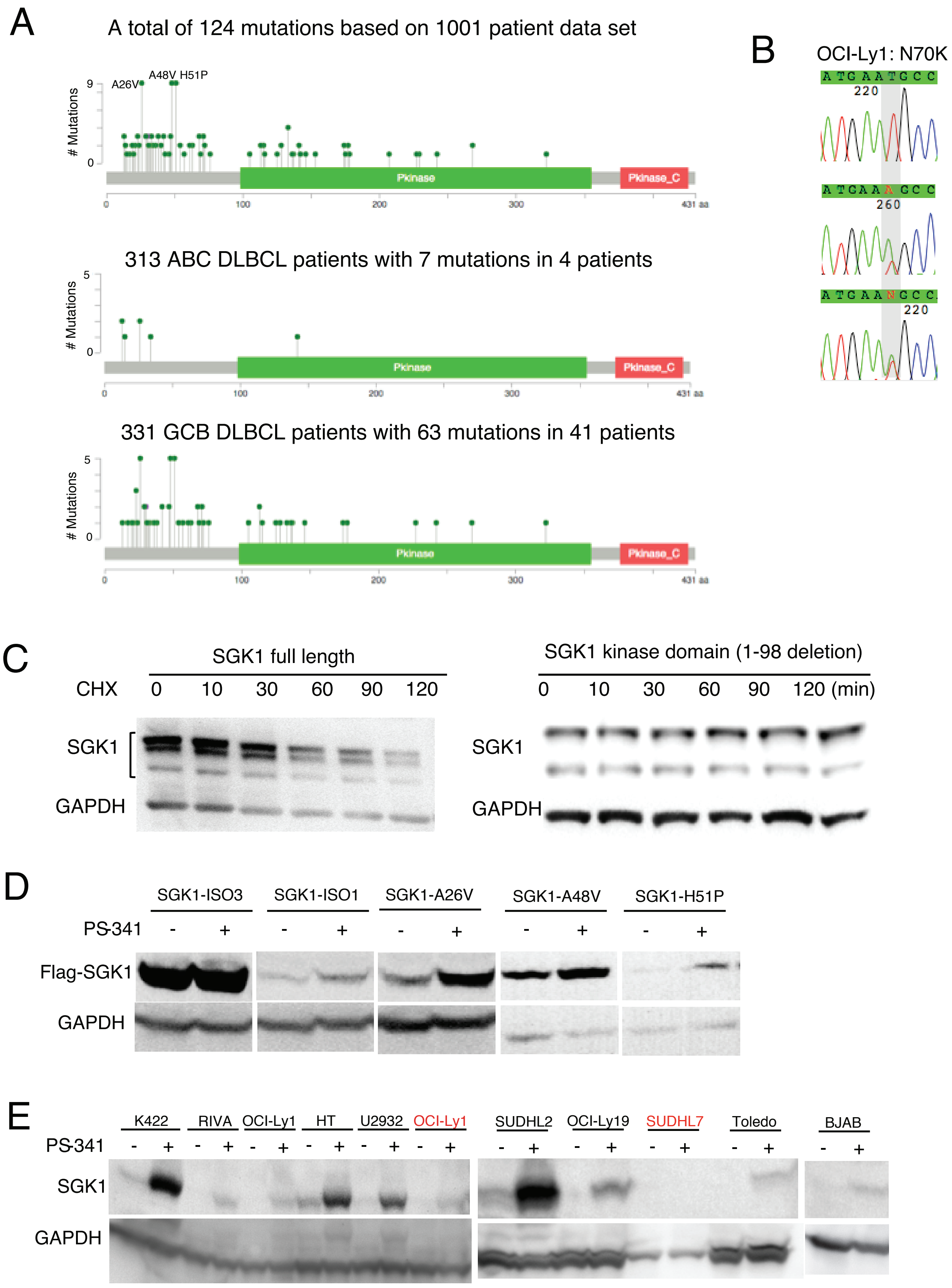
A
A total of 124 mutations based on 1001 patient data set
A26V
A48V H51P
Mutations
Pkinase
Pkinase_C
313 ABC DLBCL patients with 7 mutations in 4 patients
331 GCB DLBCL patients with 63 mutations in 41 patients
B
OCI-Ly1: N70K
C
SGK1 full length
SGK1 kinase domain (1-98 deletion)
CHX
0 10 30 60 90 120
0 10 30 60 90 120 (min)
SGK1
GAPDH
D
SGK1-ISO3
SGK1-ISO1
SGK1-A26V
SGK1-A48V
SGK1-H51P
PS-341
Flag-SGK1
GAPDH
E
K422
RIVA
OCI-Ly1
HT
U2932
OCI-Ly1
SUDHL2
OCI-Ly19
SUDHL7
Toledo
BJAB
PS-341
SGK1
GAPDH

Figure S4

Figure S4. **SGK1 mutations and SGK1 protein turnover.** (A) A total of 124 non-synonymous SGK1 mutations are detected in 1001 patient samples, most of them are detected in GCB DLBCL subtype. Data from Reddy et al., Cell 2017. (B) Sanger sequencing shows a single N70K heterogeneity mutation in OCI-Ly1 cell line. (C) Full length and N-terminal truncated (Δ1-98) SGK1 were transduced in 293T cells, and SGK1 protein levels were analyzed after cycloheximide (CHX) treatment (100 μM). (D) Full length isoform 3 (NM_001143677.1), Full length isoform 1 (NM_005627.3), N-terminal truncated (Δ1-98) and mutated SGK1 were transduced in 293T cells, and SGK1 protein levels were analyzed after PS-341 treatment (250 nM) for 4 h. (E) Immunoblot analysis of SGK1 protein levels in ABC (red) and GCB DLBCL cell lines after PS-341 treatment (250 nM) for 4 h.

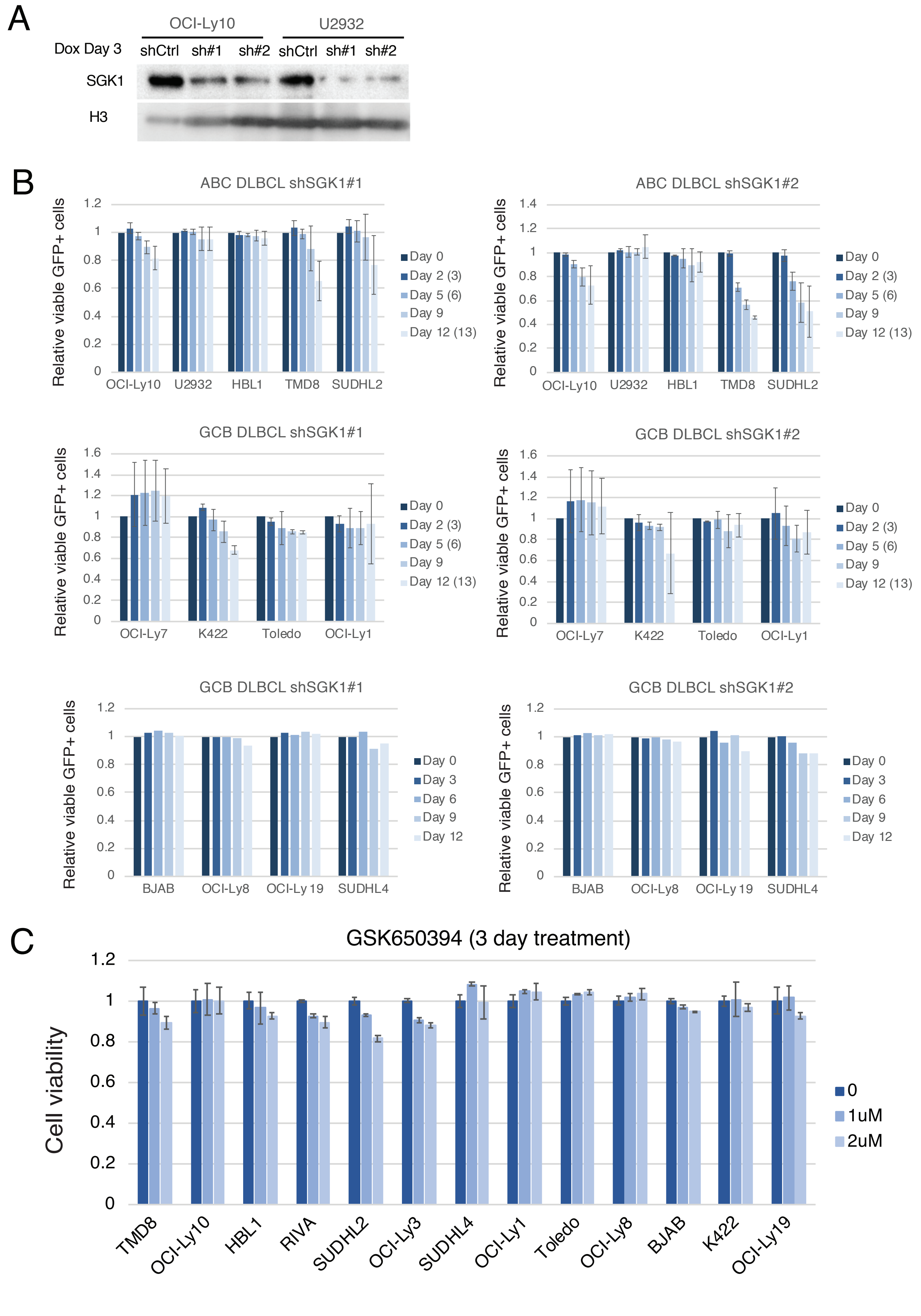
A
OCI-Ly10
U2932
Dox Day 3
shCtrl
sh#1
sh#2
SGK1
H3
B
ABC DLBCL shSGK1#1
ABC DLBCL shSGK1#2
Relative viable GFP+ cells
OCI-Ly10
U2932
HBL1
TMD8
SUDHL2
Day 0
Day 2 (3)
Day 5 (6)
Day 9
Day 12 (13)
GCB DLBCL shSGK1#1
GCB DLBCL shSGK1#2
OCI-Ly7
K422
Toledo
OCI-Ly1
BJAB
OCI-Ly8
OCI-Ly 19
SUDHL4
Day 3
Day 6
Day 12
C
GSK650394 (3 day treatment)
Cell viability
TMD8
OCI-Ly10
HBL1
RIVA
SUDHL2
OCI-Ly3
SUDHL4
OCI-Ly1
Toledo
OCI-Ly8
BJAB
K422
OCI-Ly19
0
1uM
2uM

Figure S5

Figure S5. Inhibition of SGK1 by shRNAs and the inhibitor GSK650394. (A) Immunoblot analysis of SGK1 protein in OCI-Ly10 and U2932 after SGK1 knock-down by 3 d DOX induction of shCtrl, shSGK1#1 and shSGK1#2. (B) Flow cytometric analysis of GFP positive cells after SGK1 knockdown with doxycycline induction of shSGK1#1 and shSGK1#2 for 12 days. Data were normalized to shCtrl experiments. Data indicate mean ± SE of triplicates. (C) CellTiter-Glo™ Luminescent Cell Viability Assay of DLBCL cell lines after 3 d treatment with the SGK1 inhibitor GSK650394 by the indicated concentrations. Data indicate mean ± SE of triplicates.

A

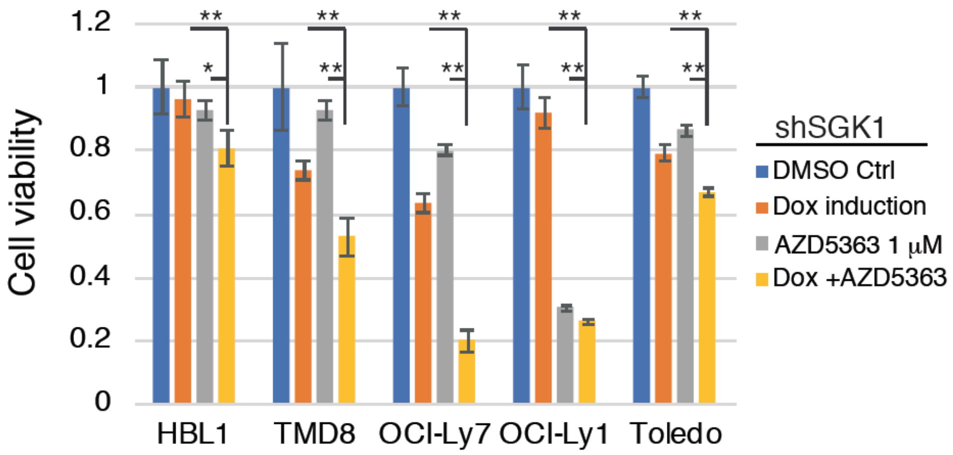

B

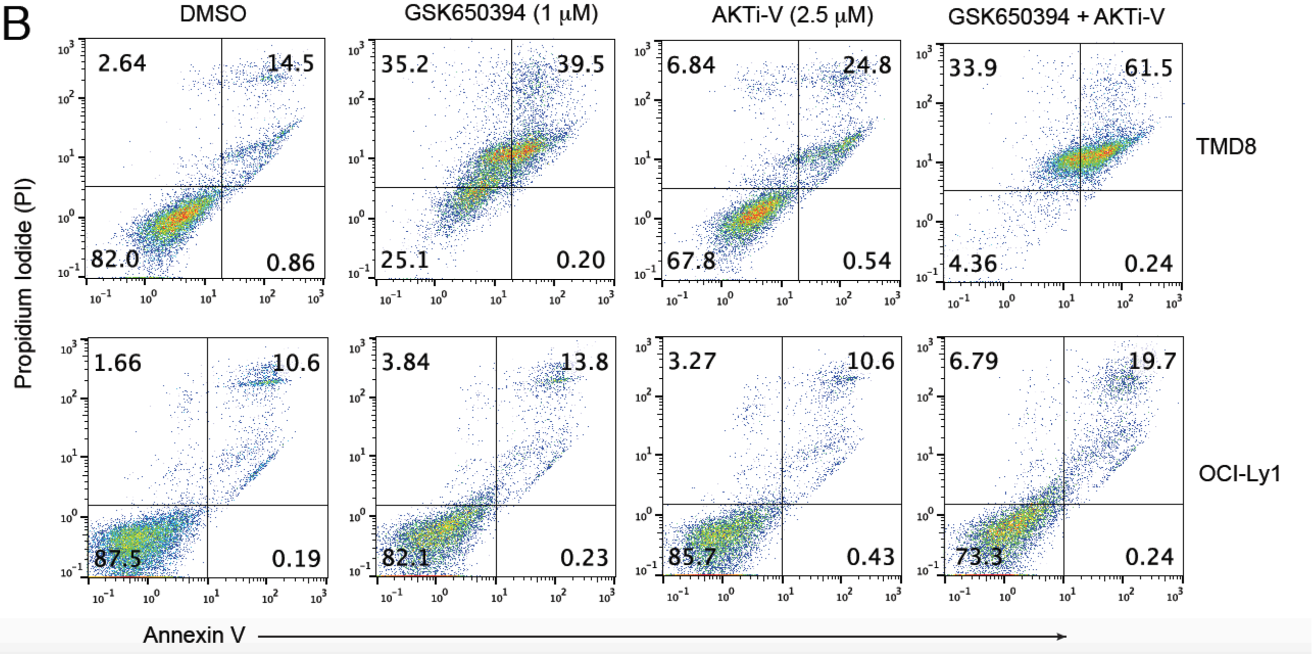

Figure S6. Inhibition of SGK1 by shRNA sensitizes DLBCL cells to the AKT inhibitor AZD5363 (A) CellTiter-Glo™ Luminescent Cell Viability Assay of HBL1, TMD8, OCI-Ly7, OCI-ly1 and Toledo cells after 5 d induction with SGK1 knockdown (shSGK1#2) or 6 d treatment of AZD5363 (1 μM), or both. Data indicate mean ± SE of triplicates. *, $p < 0.05$; **, $p < 0.01$. (B) Flow cytometric analysis of cell apoptosis by Annexin V and propidium iodide (PI) staining in TMD8 and OCI-Ly1 cells when treated with GSK650394 and AKTi-V alone or both.
